# Supplementary figures and images for: A high-resolution, easy-to-build light-sheet microscope for subcellular imaging
Source: eLife. 2026 Feb 5;14:RP106910. doi: 10.7554/eLife.106910 (PMC12875610; doi:10.7554/eLife.106910)

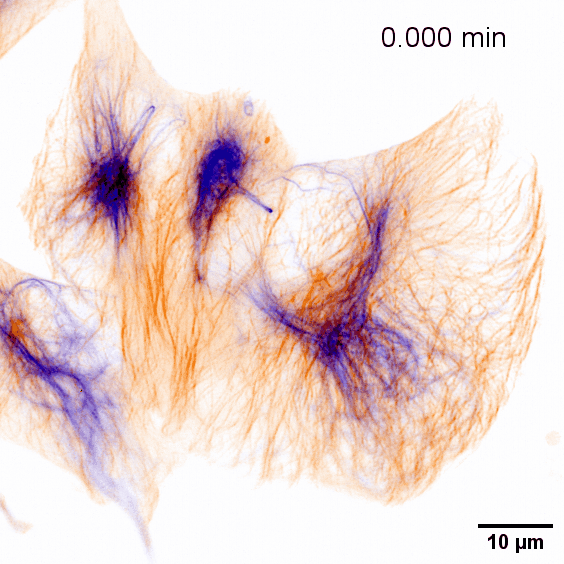

Supplement: Supplementary file 1 [file elife-106910-animation1.gif]
