# Supplementary material for: A high-resolution, easy-to-build light-sheet microscope for subcellular imaging
Source: eLife. 2026 Feb 5;14:RP106910. doi: 10.7554/eLife.106910 (PMC12875610; doi:10.7554/eLife.106910)
Supplement: Supplementary file 1. [file elife-106910-supp1.docx]

| **Name** | **Manufacturer** | **Description** | **Qty** | **Approximate Cost** |
| --- | --- | --- | --- | --- |
| **Shared Equipment** | | | | |
| TG16-BASIC | ASI | Tiger Controller – 16 Bay System | 1 | $6,350 |
| PCIe-6738 | NI | Data Acquisition Card | 1 | $2,200 |
| SCB-68A | NI | Noise Rejecting Terminal Block | 1 | $500 |
| SHC68-68-A2 | NI | Test Cable Assembly | 1 | $250 |
| 784-736-02R | TMC | 36x60x18” Performance Series Optical Top | 1 | $6,275 |
| 14UD-42X-24 | TMC | Ultradamp Vibration Isolation with Casters | 1 | $9,300 |
| SX6300 | Colfax International | Colfax SX6300 Workstation | 1 | $6,800 |
|  | | | | |
| **Detection Path** | | | | |
| LS-100-AMCCH | ASI | 100 mm Linear Focusing Stage, 16 TPI | 1 | $2,250 |
| TGDCM2 | ASI | 2-Axis Stage Control Card | 1 | $1,050 |
| C60-EXT-15 | ASI | 15 mm Tube Extension | 1 | $75 |
| RAO-0051 | ASI | M32x0.75 Threaded Sleeve | 1 | $150 |
| FW-0002-8 | ASI | 8-Position Filter Wheel | 1 | $3,500 |
| FW-C-MNT-K1 | ASI | Filter Wheel to MIM Adapter Kit | 1 | $400 |
| C60-TUBE-400 | ASI | 400 mm Achromatic Tube Lens | 1 | $875 |
| TGFW | ASI | Filter Wheel Control Card | 1 | $1,150 |
| C13440-20CU | Hamamatsu | ORCA Flash4.0 V3 | 1 | $16,500 |
| OH-CAMRA-1007 | Hamamatsu | Firebird CamLink Board | 1 | $2,000 |
| N25X-APO-MP | Thorlabs | Nikon 25x NA 1.1 Detection Objective | 1 | $33,810 |
| 445/20-25nm | Semrock | Brightline Bandpass filter | 1 | $375 |
| 529/24nm | Semrock | Brightline Bandpass filter | 2 | $375 |
| 605/15-25nm | Semrock | Brightline Bandpass filter | 1 | $375 |
| 676/29-25nm | Semrock | Brightline Bandpass filter | 1 | $375 |
|  | | | | |
| **Sample Positioning** | | | |  |
| LS-5012 | ASI | Breadboard Adapter | 1 | $160 |
| LS-5013 | ASI | Right Angle Bracket | 1 | $250 |
| DV-6010-C | ASI | Dovetail Mount Pair | 1 | $230 |
| LS-50-AMCLLS | ASI | 50 mm Linear Stage with Stainless Steel Slide | 1 | $2,425 |
| LS-100-AMERL | ASI | 100 mm Linear Stage, 16 TPI, Extended, Right | 1 | $3,100 |
| LS-50-AMELL | ASI | 50 mm Linear Stage | 1 | $2,250 |
| TGADEPT | ASI | Piezo Control Card | 1 | $1,500 |
| HS1.100 | PiezoConcept | Piezo Concept 100 Micron Piezo Stage | 1 | $4,100 |
| ADAPTHS1BB | PiezoConcept | Adapter Plate for PiezoConcept HS1 | 1 | $120 |
| Angle Bracket Adapter | Xometry | Angle adapter for mounting piezo at an angle | 1 | $592.58 |
|  | | | | |
| **Illumination Path** | | | | |
| L4CC | Oxxius | Multicolor Laser Source | 1 | 25,000 |
| P3-405B-FC-1 | Thorlabs | Fiber Cable Single Mode FC/APC | 1 | $120 |
| CFC11A-A | Thorlabs | Fiber Collimator | 1 | $370 |
| Polaris-K1XY | Thorlabs | Kinematic Mount for Fiber Laser Collimator | 1 | $1375 |
| AC254-030-A-ML | Thorlabs | Achromatic Lens f=30mm (L1) | 1 | $125 |
| AC254-080-A-ML | Thorlabs | Achromatic Lens f=80mm (L2) | 1 | $115 |
| ACY254-075-A | Thorlabs | Achromatic Cylindrical Lens f=75mm (L3) | 1 | $445 |
| AC254-250-A-ML | Thorlabs | Achromatic Lens f=250mm (L4) | 1 | $90 |
| PF10-03-P01 | Thorlabs | 1” Protected Silver Mirror | 1 | $55 |
| TL20X-MPL | Thorlabs | Illumination Objective | 1 | $4820 |
| VA100CP | Thorlabs | Rectangular Aperture | 1 | $315 |
| IDA12 | Thorlabs | Circular Aperture Iris | 1 | $60 |
| Polaris-P150 | Thorlabs | 1” Diameter Polaris Mounting Post, 1.5” Length | 1 | $45 |
| Polaris-P2 | Thorlabs | 1” Diameter Polaris Mounting Post, 2” Length | 1 | $45 |
| Polaris-P3 | Thorlabs | 1” Diameter Polaris Mounting Posts, 3” Length | 3 | $45 |
| Polaris-P075* | Thorlabs | 1” Diameter Polaris Mounting Post, 0.75” Length | 1 | $45 |
| Polaris-P225* | Thorlabs | 1” Diameter Polaris Mounting Post, 2.25” Length | 1 | $45 |
| Polaris-P250* | Thorlabs | 1” Diameter Polaris Mounting Post, 2.5” Length | 2 | $45 |
| Polaris-MA45 | Thorlabs | Polaris 45 Degree Adapter for Mirror Mount | 1 | $50 |
| Polaris-B1S | Thorlabs | Polaris Flexure-Closed Lens Mount | 3 | $110 |
| Polaris-1XY | Thorlabs | Polaris XY Translation Mount for Illumination Objective | 1 | $1020 |
| Polaris-K1S4 | Thorlabs | Polaris 1” Mirror Mount | 1 | $185 |
| SM1A12 | Thorlabs | Thread Adapter for Illumination Objective into Polaris-1XY | 1 | $25 |
| 6SC04KA040-01Y | Novanta | 1-Axis 4 kHz Resonant Galvo and Servo | 1 | $3,700 |
| Galvo Holder | Protolabs | Holder for Galvo | 1 | $400 |
| TGPOW-12-3 | ASI | Galvo Low Noise Power Supply +12V 3A | 1 | $950 |
| RSP1 Adapter | Xometry | Custom Adapter for Thorlabs RSP1 to Polaris Posts | 1 | $80 |
| VA100CP Adapter | Xometry | Custom Adapter for Thorlabs VA100CP to Polaris Posts | 1 | $80 |
| CP02 Adapter | Xometry | Custom Adapter for Thorlabs CP02 to Polaris Posts | 1 | $80 |
| Illumination Path Baseplate | Xometry | Baseplate for Illumination Path | 1 | $1000 |
|  |  |  |  |  |
| **Live-Cell Imaging** |  |  |  |  |
| Custom Sample Chamber | Xometry | Custom Designed Live-Cell Sample Chamber | 1 | $530 |
| 1797N11 | McMaster Carr | Benchtop Autotuning Temperature Controller, Type J Thermocouple, 1 Bay | 1 | $835 |
| 1797N103 | McMaster Carr | Benchtop Autotuning Temperature Controller, Type J Thermocouple, 2 Bay | 1 | $1390 |
| 5586N11 | McMaster Carr | 3" Dual Thermocouple 1/8" Diameter Probe, Type J | 1 | $120 |
| 5843N12 | McMaster Carr | 1" Threaded Thermocouple 1/4"-20 Threading | 1 | $100 |
| 35765K373 | McMaster Carr | 2x5" Adhesive Backed Heat Sheet | 1 | $80 |
| 35475K362 | McMaster Carr | 5x1" Ultrathin Heat Sheet | 1 | $120 |
| 35765K367 | McMaster Carr | 6x1" Adhesive Backed Heat Sheet | 1 | $75 |

Supplementary Table 1. Detailed equipment list. Prices are approximate and subject to change. *Indicates that the part was custom ordered from Thorlabs.
