# Supplementary material for: A high-resolution, easy-to-build light-sheet microscope for subcellular imaging
Source: eLife. 2026 Feb 5;14:RP106910. doi: 10.7554/eLife.106910 (PMC12875610; doi:10.7554/eLife.106910)
Supplement: Supplementary file 2. [file elife-106910-supp2.docx]

| **Group** | **Approximate Cost** |
| --- | --- |
| Illumination Path | $41,195 |
| Sample Positioning | $14,727 |
| Detection Path | $63,635 |
| Shared Equipment | $31,675 |
| Live-Cell Imaging | $3250 |
| Total: | $154,482 |

Supplementary Table 2. Approximate cost.
