# Supplementary material for: A high-resolution, easy-to-build light-sheet microscope for subcellular imaging
Source: eLife. 2026 Feb 5;14:RP106910. doi: 10.7554/eLife.106910 (PMC12875610; doi:10.7554/eLife.106910)
Supplement: Supplementary file 3. — All analog and digital connections were made using a National Instruments SCB-68A shielded terminal block. [file elife-106910-supp3.docx]

| **Component** | **Analog/Digital** | **Ground Pin** | **Active Pin** |
| --- | --- | --- | --- |
| Resonant Galvo | Analog | 14 | 13 |
| Piezo Motor | Analog | 11 | 10 |
| Camera | Digital | 43 | 9 |
| Fiber Laser (FL) Output Shutter | Digital | 39 | 5 |
| FL λ = 405 nm AO Port | Analog | 55 | 21 |
| FL λ = 405 nm IO Port | Digital | 42 | 8 |
| FL λ = 488 nm AO Port | Analog | 18 | 52 |
| FL λ = 488 nm IO Port | Digital | 41 | 7 |
| FL λ = 561 nm AO Port | Analog | 20 | 54 |
| FL λ = 561 nm IO Port | Digital | 41 | 40 |
| FL λ = 638 nm AO Port | Analog | 50 | 16 |
| FL λ = 638 nm IO Port | Digital | 39 | 6 |

Supplementary Table 3. Electrical pinouts used on National Instruments PCIe-6738 data acquisition card. All analog and digital connections were made using a National Instruments SCB-68A shielded terminal block.
