# Supplementary material for: A high-resolution, easy-to-build light-sheet microscope for subcellular imaging
Source: eLife. 2026 Feb 5;14:RP106910. doi: 10.7554/eLife.106910 (PMC12875610; doi:10.7554/eLife.106910)
Supplement: Supplementary file 4. [file elife-106910-supp4.docx]

| **Exposure Time (ms)** | **Average Frame Rate (Hz)** |
| --- | --- |
| 10 | 62.5 |
| 50 | 17.6 |
| 100 | 9.2 |
| 200 | 4.8 |

Supplementary Table 4. Acquisition performance for a 50 µm z-stack acquired at 0.25 µm step size (200 frames; 2048 × 512 pixels per frame). The mean inter-frame dead time was 7.25 ms, of which ~1 ms arose from piezo stepping; the remainder was dominated by camera readout.
