# Supplementary material for: A high-resolution, easy-to-build light-sheet microscope for subcellular imaging
Source: eLife. 2026 Feb 5;14:RP106910. doi: 10.7554/eLife.106910 (PMC12875610; doi:10.7554/eLife.106910)
Supplement: Supplementary file 5. [file elife-106910-supp5.docx]

| **Experience** | **Illumination Path Assembly** | **Full System Assembly** | **Fine Alignment** | **Imaging & Validation** |
| --- | --- | --- | --- | --- |
| **Novice** | ~1-3 hours | 1-4 Days | 1 Week | 2 Weeks |
| **Moderate** | ~1-3 hours | 1-4 Days | 1 Week | 1 Week |
| **Expert** | ~1 hour | 1 Day | 1-2 Days | 1 Week |

Supplementary Table 5. Approximate time considerations based on user experience level. A novice is defined as someone entirely new to optical systems, with no prior experience in their operation or alignment. A moderate user has some prior experience operating or using optical systems but limited experience assembling or aligning them. An expert user has substantial experience designing, building, and aligning custom optical systems and is therefore expected to complete setup and validation tasks more efficiently.
